# Acid-base assessment of post-parturient German Holstein dairy cows from jugular venous blood and urine: A comparison of the strong ion approach and traditional blood gas analysis

**DOI:** 10.1371/journal.pone.0210948

**Published:** 2019-01-16

**Authors:** Tanja Gärtner, Veit Zoche-Golob, Stefanie Redlberger, Petra Reinhold, Karsten Donat

**Affiliations:** 1 Thuringian Animal Health Fund, Cattle Health Service, Jena, Thuringia, Germany; 2 Institute of Molecular Pathogenesis, Friedrich-Loeffler-Institut (Federal Research Institute for Animal Health), Jena, Thuringia, Germany; University of Illinois, UNITED STATES

## Abstract

Evaluating acid-base status is important for monitoring dairy herd health. In a field study, we aimed to compare the acid-base status measured by net acid-base excretion (NABE) in urine with results of venous blood analysis in clinically healthy, but possibly metabolically burdened cows in their transition period. For this, we sampled blood from the jugular vein and urine from 145 German Holstein cows within 1 to 76 days post-partum. In blood, the metabolic parameters non-esterified fatty acids (NEFA) and β-hydroxybutyrate (BHB), as well as numerous parameters of the acid-base status were measured. The traditional approach, based on bicarbonate concentration, base excess (BE) and anion gap (AG), was compared to the strong ion approach variables, e.g. acid total (A_tot_), measured strong ion difference (SID_m_), strong ion gap (SIG), and unmeasured anions (XA), respectively. Results of both approaches were set against the outcome of urine analysis, i.e. the NABE, base-acid ratio and pH of urine, in a cluster analysis, which provided 7 moderately stable clusters. Evaluating and interpreting these 7 clusters offered novel insights into the pathophysiology of the acid-base equilibrium in fresh post-partum dairy cows. Especially in case of subclinical acid-base disorders, the parameters of the strong ion difference theory, particularly SID_m_, A_tot_ and SIG or XA, provided more in-depth information about acid-base status than the traditional parameters BE, bicarbonate or AG in blood. The acid-base status of fresh cows with protein aberrations in blood could be differentiated in a much better way using the strong ion approach than by traditional blood gas analysis or by the measurement of urinary excretion. Therefore, the strong ion approach seems to be a suitable supplement for monitoring acid-base balance in dairy cattle.

## Introduction

Monitoring the acid-base status in cattle is an important diagnostic tool in dairy herd health management. Inappropriate feeding is one of the major causes of ruminants’ predisposition to metabolic imbalances leading to reduced health and performance in dairy herds [1, 2]. Monitoring such metabolic disturbances can include analysis of blood, urine, milk or ruminal fluid [2, 3].

The traditional approach for evaluating the acid-base status in peripheral blood is bicarbonate-based with determination of base excess and anion gap [4, 5]. The anion gap (AG) was established for detecting unidentified strong ions, indicating that an increase in AG shows acidosis induced by an increase in unmeasured anions [6]. Thereby the AG depends on albumin concentration [7]. The traditional approach is recommended for use only when serum protein concentration is not affected [8, 9].

In the 1980s, Stewart [10] defined a new approach for assessing the acid-base equilibrium by combining the three constraints of electroneutrality, dissociation equilibrium, and conservation of mass. Stewart proposed that plasma pH is determined by three independent factors, i.e. partial pressure of CO_2_ (pCO_2_), strong ion difference (SID), and acid total (A_tot_) [4, 5]. SID represents the net strong ion charge from plasma and is defined as the difference between the quantitatively important measurable strong cations (e.g. sodium, potassium, calcium and magnesium) and anions (e.g. chloride and lactate) in plasma [10]. A_tot_ represents the total plasma concentration of non-volatile weak buffers and involves the plasma concentrations of albumins, globulins and phosphate [10], but Stewart’s approach provides no valid method to determine A_tot_ in cattle [11].

After Stewart described this theoretical basis, his strong ion model was applied to many species expecting novel insights into the pathophysiology of mixed acid-base disorders [7, 12, 13]. Furthermore, it was extended to detect unmeasured strong anions (XA) [13] and was recommended for use in cattle by considering globulin concentrations in bovine serum [14, 15]. Constable reduced Stewart’s complex equations to a simplified approach for clinical assessment [16] and provided accurate values for A_tot_ in bovine plasma [14]. He supplied the calculation of the strong ion gap (SIG) which included A_tot_ and, therefore, allowed a better estimation of the unmeasured strong ion concentration in plasma than the anion gap. As bovine globulin is negatively charged, calculations were given based on serum concentrations of albumin (A_tot(Alb)_, SIG_Alb_) or total protein (A_tot(Prt)_, SIG_Prt_) respectively [14]. The SIG appears to be the central advantage of Stewart´s theory.

In urine, the measurement of the net acid-base excretion (NABE), established by Jørgensen [17] and Kutas [18] and refined by Lachmann [2], has been widely used in bovine medicine and has been proved to provide a sensitive and appropriate method to evaluate the acid-base status in cattle in general practice [1, 3, 19]. Thus, urinary NABE is an important component of the metabolic profile tests in dairy herds.

None of the methods described above have proved to be superior in detecting early or subclinical acid-base disturbances in clinically healthy cows. Furthermore, acid-base assessment in urine and the strong ion approach in blood had, thus far, not been simultaneously applied to dairy cows in field studies. By doing this, the objective of this study was to obtain deeper insights into the pathophysiology of acid-base imbalances in metabolically burdened albeit clinically healthy dairy cows by combining urine analysis with the two different methods of blood analysis. Thus, the diagnostic value of NABE resulting from urine analysis was compared to the outcomes of acid-base variables in blood resulting from the traditional approach involving determination of bicarbonate and base excess and the strong ion model, respectively. Our hypothesis was that subclinical metabolic imbalances could be identified in a better way by combining urine and blood analyses, and by using the strong ion approach instead of the traditional approach to assess the acid-base equilibrium in blood.

## Animals, materials and methods

### Ethics statement

Clinical examinations and collection of biological specimens were performed within the framework of the cattle health monitoring program of the Thuringian Animal Health Service, where cows of the herds under contract are routinely supervised and monitored by veterinarians. Every effort was made to minimize suffering during the sampling of blood and urine. Since it was not possible to collect arterial blood within the framework of this permit only venous blood was sampled for further analysis. The competent authority for research ethics approval (Thuringian State Office for Consumer Protection, Department 2 Animal Welfare) granted a formal waiver of ethics approval (approval number 2684-04-15-TSK-18-001).

### Animals and study design

A total of 145 German Holstein dairy cows from 13 farms in Thuringia were included in the study. The cows showed no overtly apparent clinical symptoms of disease and were either fresh cows 1–15 days post calving (n = 129) or high lactating cows, being 43–76 days in milk (DIM) (n = 16). Obviously diseased animals like downer cows or cows with rectal body temperature > 40°C were excluded from the study.

In parallel to sampling, the rectal temperature was measured, body condition score (BCS) according to Edmunson et al. [20] was assigned, and the rumen fill, scored on a scale of 1 to 3 (1 –nearly empty; 2 –moderately-filled; 3 –well-filled) was noted. Blood and urine were obtained from each animal on one occasion, with samples collected immediately after each other and evaluated as a pair. Blood was collected anaerobically from the jugular vein into 10 ml vacuumized serum tubes for biochemical analysis and into 2 ml polypropylene syringes with lyophilized electrolyte-balanced heparin for blood gas and electrolyte analysis. Urine samples were collected anaerobically in 13 ml tubes using a sterile bladder catheter (Breslau model, material: stainless steel).

### Urine analysis

Without any further processing, the urine samples were stored at -20°C until analysis. The concentrations of sodium (cNa^+^ (u)), potassium (cK^+^ (u)), calcium (cCa^2+^ (u)) and chloride (cCl^-^ (u)) were obtained by indirect ion-selective potentiometry. Magnesium (cMg (u)) was measured with an automatic analyzer (UniCel DxC 600, Beckman Coulter; timed endpoint method). The pH of urine (pH (u)) was assessed by pH-meter at room temperature (22±2 °C, air-conditioning). Additionally, the net acid-base excretion (cNABE (u)) with base excess (cBE (u)), acid excess (cAE (u)), the concentration of ammonium (cAmm (u)) and the base-acid ratio (BAR (u)) were determined by the titrimetric method as described by Lachmann and Schäfer [2]. Shortly, 10 ml of urine were titrated first with hydrochloric acid to reduce pH to 4.0 whereas the required amount of acid corresponded to cBE. Second, the sample was titrated with sodium hydroxide to raise pH again to 7.0, and the used amount of lye resulted in cAE. Last, formyl aldehyde was used to precipitate ammonium ions, pH was titrated with lye to 7.0 again in order to assess the cAmm in urine. After this, cNABE and BAR were calculated by the following Eqs 1 and 2:

cNABE=cBE–(cAE+cAmm) (1)

BAR=cBE/(cAE+cAmm) (2)

### Blood analysis

Immediately after collection, the heparinized blood samples were kept on cooled packs, i.e. at temperatures below 8°C. They were analyzed within 2 and 4 hours using a combined blood gas/electrolyte analyzer (ABL-series, Radiometer) as described previously by Ostermann et al. [21] and Redlberger et al. [22]. The venous blood pH (pH (v)), the partial pressure of CO_2_ (pCO_2_ (v)) and the concentration of total hemoglobin (ctHb) as well as the plasma ion concentrations of sodium (cNa^+^), potassium (cK^+^), ionized calcium (cCa^2+^) and chloride (cCl^-^) were measured by ion-selective potentiometry, and glucose (cGlucose) and L-lactate (cL-Lactate) by enzymatic electrodes. By assuming 100% dissociation, a charge of +1 or +2, respectively, was assumed for the cations and -1 for chloride and L-lactate.

The serum samples were prepared by centrifugation at room temperature, and were stored afterwards at -20°C until analysis. An automatic analyzer (UniCel DxC 600, Beckman Coulter) was used for measuring the serum concentrations of non-esterified fatty acids (cNEFA, enzymatic colorimetric method), β-hydroxybutyrate (cBHB, enzymatic method), urea (cUrea, enzymatic method), total bilirubin (cBilirubin, Jendrassik-Grof), cholesterol (cCholesterol, timed endpoint method), inorganic phosphorous (cPi, timed endpoint method) and total magnesium (cMg (b), timed endpoint method), and the activity of glutamate-dehydrogenase (aGLDH, IFCC method) photometrically. A net charge of +1.38 for magnesium (cMg^2+^ (b)) was assigned by assuming 69% dissociation [23]. For determining total protein, albumin and globulin concentrations as well as the spectra of globulins and the albumin globulin ratio (AGR), capillary electrophoresis was performed (Capillarys 2, Sebia).

### Calculated acid-base variables

Using standard algorithms integrated in the software of the blood gas analyzer, blood pH and pCO_2_ (v) were corrected for rectal body temperature (BT) assessed immediately before blood collection. In addition, the traditional variables standard bicarbonate (cHCO_3_^-^ (st)), actual base excess (cBase), standard base excess (cBase (Ecf)), and the hematocrit (Hct) were calculated by standard software as described by Siggaard Andersen and colleagues [24–26] on the basics of the Henderson-Hasselbalch equation [27, 28] and the Van Slyke Equation [29]. Further parameters and variables used for the interpretation of the traditional and modern acid-base concepts are given in Table 1.

**Table 1:** Description of applied parameters and variables of acid-base equilibrium in blood.

Parameters / Variables	Units	Values / Formulae	References
S	mmol/L * (mm Hg)^-1^	0.0307	[30]
pK_1_´		6.12	[31]
cHCO_3_^-^	mmol/L	S * pCO_2_ * 10^(pH-pK1´)^	[28]
AG	mEq/L	(cNa^+^ + cK^+^)—(cCl^-^ + cHCO_3_^-^)	[32, 33]
SID_m3_	mEq/L	(cNa^+^ + cK^+^)—cCl^-^	[11, 21]
SID_m4_	mEq/L	(cNa^+^ + cK^+^)–(cCl^-^ + cL-Lactate)	[11, 21]
SID_m5_	mEq/L	(cNa^+^ + cK^+^ + cCa^2+^)–(cCl^-^ + cL-Lactate)	[11, 21]
A_tot(Alb)_	mEq/L	Alb[g/L] * 0.76	[14]
A_tot(Prt)_	mEq/L	TP[g/L] * 0.36	[14]
pK_a_	mEq/L	7.06	[14]
SIG_(Alb)_	mEq/L	A_tot(Alb)_ /(1+10^(pKa-pH)^)—AG	[14]
SIG_(Prt)_	mEq/L	A_tot(Prt)_ /(1+10^(pKa-pH)^)—AG	[14]
Albumin charge	mEq/L	0.141 * cAlbumin * (pH– 5.42)	[15, 34]
Globulin charge	mEq/L	0.04 * (cProtein total–c Albumin) * (pH– 5.58)	[15, 34]
Phosphate charge	mEq/L	cPi (b) * (0.309 * pH– 0.469)	[15, 34]
XA	mEq/L	cNa^+^(b)+ cK^+^(b) + cCa^2+^(b) + cMg^2+^(b) − cCl^-^(b)- cHCO_3_^-^ − (Albumin charge) − (Globulin charge) − (Phosphate charge)	[13, 15]

S = solubility of carbon dioxide, pK_1_ = negative logarithm of the dissociation constant of carbonic acid, cHCO_3_^-^ = actual bicarbonate concentration, AG = Anion gap, SIDm = measured strong ion difference, A_tot_ = Acid total, pK_a_ = negative logarithm of dissociation constant of plasma nonvolatile weak acids, SIG = strong ion gap, XA = unmeasured anions

### Data analysis

To identify groups of cows with a similar physiological status, a hierarchical cluster analysis was conducted. Due to the limited number of observations, we decided to use, at most, one variable per ten observations as a segmentation base. These variables were selected using a physiological context. As the main dependent parameter of the acid-base balance in blood, pH(v)_BT_ was considered in cluster building. In addition, the independent variables A_tot(Alb)_ and SID_m5_ were chosen, as well as AGR and hematocrit, with the latter allowing for the identification of the dilutional effects or hemoconcentration. Additionally, cBHB, cNEFA and SIG_Prt_ were included for presenting the unmeasured ion fractions. The urine variables cNa (u), Cl (u) and BAR (u), which influenced and provided the best evaluation of acid-base excretion, completed the selection for cluster determination.

Pairwise correlation analyses were performed for all quantitative variables using Pearson’s correlation coefficient, and no pair of highly correlated variables (|rho| < 0.75) was included in the cluster analysis. All variables of the segmentation base were standardized and the Euclidean distance was used to calculate the distance matrix. The cluster agglomeration was performed using Ward’s minimum variance method. According to the guidelines proposed by Hennig [35], the number of meaningful clusters was determined by employing the following criteria:

- the interpretability and discernibility of the clusters from a physiological perspective, and
- the internal validity of the clustering assessed using silhouette plots [36].

Heatmaps and boxplots were compiled to facilitate the interpretation of the clusters and to compare them. After a preliminary decision on the number of clusters, the clusters were compared with respect to all the variables for the final decision. The data analysis was conducted in R, version 3.3.1 [37] using the additional package cluster, version 2.0.4 [38].

## Results

**Fig 1:**
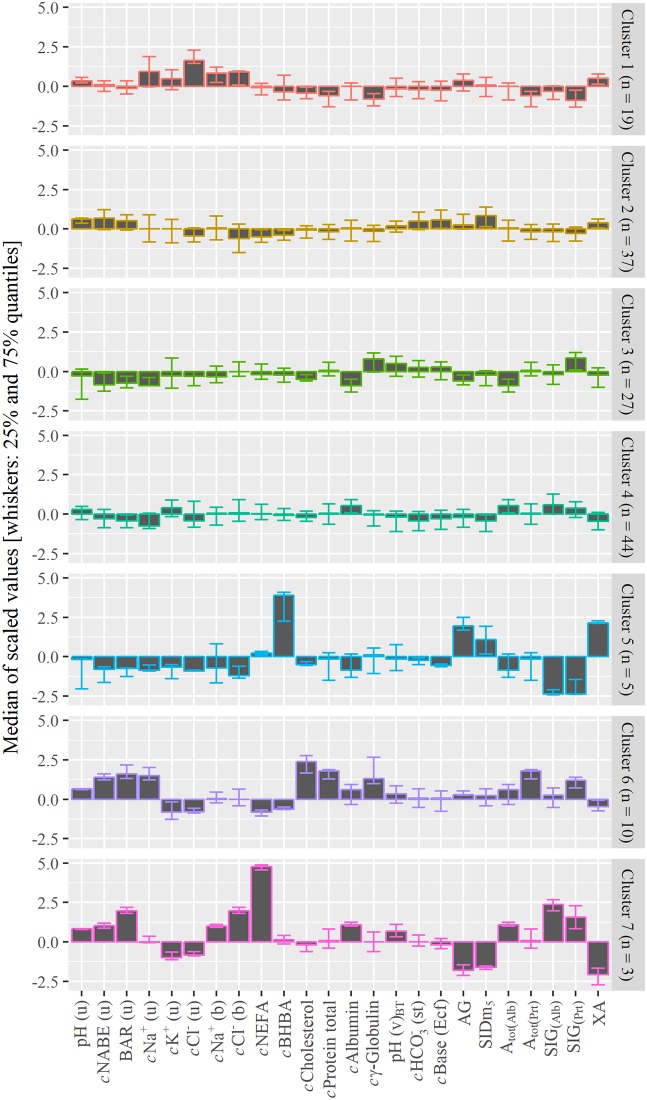
Evaluated parameters of urine and blood and their distribution in the 7 clusters. Centered and scaled medians (filled boxes) with the 50 percent interquartile range (whiskers).

The cluster analysis provided 7 moderately stable clusters (Fig 1). For all measured and calculated parameters, the medians as well as the first and third quartiles are given in S1 Table. For a selection of parameters, their standardized deviations per cluster from the overall median are shown in Fig 1. The main results of the common parameters of the acid-base equilibrium in blood and urine are given in Table 2. As shown in Table 3, the animals and the farms were not allocated equally to the 7 clusters. The results of the clinical examination per cluster and the medians of the assessed clinical parameters are also represented in Table 3.

**Table 2:** Main results of different approaches to evaluate acid base equilibrium in the generated clusters.

Results of parameters		Cluster							Reference values
		1 n = 19	2 n = 37	3 n = 27	4 n = 44	5 n = 5	6 n = 10	7 n = 3	
**Urine**	pH	Ø		↓	↓	↓	Ø	↑	8.17–8.35^a^
	NABE	Ø		↓↓	↓	↓↓	↑	Ø	106–210^a^
	BAR	↓		↓↓	↓	↓↓	↑	↑	2.3–3.6^a^
Interpretation of urine results		tends to acidosis		strong acidosis	acidosis	strong acidosis	alkalosis	alkalosis	
**Blood gases: traditional approach**	pH	Ø		Ø	Ø	Ø	Ø	Ø	7.38–7.40^a^
	HCO_3_^-^ (st)	↓		Ø	↓	↓	Ø	Ø	27.0–28.8^a^
	SBE	↓		Ø	↓	↓	Ø	↓	4.3–6.5^a^
	AG	Ø		↓	↓	↑	Ø	↓↓	13.5–15.3^a^
Interpretation of traditional blood gas approach		acidosis		Ø	acidosis	acidosis, increased unidentified anions	Ø	(acidosis) increased unidentified cations	
**Blood gases: strong ion approach**	SID_m5_	↓		↓	↓	Ø	Ø	↓↓	42.6–45.5^a^
	A_tot(Alb)_	Ø		↓	Ø	↓	↑	↑↑	21.4–25.5^a^
	A_tot(Prt)_	Ø		Ø	Ø	Ø	↑↑	Ø	24.5–27.2^a^
	SIG_(Alb)_	Ø		Ø	↑	↓↓	Ø	↑↑	0.5–3.4^a^
	SIG_(Prt)_	↓		↑	↑	↓↓	↑	↑↑	2.4–4.7^a^
	XA	Ø		Ø	Ø	↑↑	Ø	↓↓	7.4–8.8^a^
Interpretation of strong ion approach		SID-acidosis		SID-acidosis	SID-acidosis	increased unidentified anions	A_tot_ -acidosis	SID-acidosis,	
								increased unidentified cations	
**Other meta-bolism**	NEFA	↑	Ø	↑	↑	↑	(↓)	↑↑↑	< 0.62^b^
	BHBA	Ø	Ø	Ø	Ø	↑↑	(↓)	(↑)	< 1200^b^
	Cholesterol	(↓)	Ø	(↓)	Ø	(↓)	(↑)	Ø	> 1.5^b^
	Albumin	Ø	Ø	↓	(↑)	↓	(↑)	(↑)	30–39^b^
	γ-globulins	(↓)	Ø	(↑)	Ø	Ø	↑	Ø	16.4–30.4^b^
Interpretation of other metabolism		reduced feed intake	normal	reduced feed intake	(normal)	keto-acidosis	extended feed intake	extended fat mobilization	

NABE = net acid base excretion, BAR = base acid ratio, HCO_3_^-^ (st) = standard bicarbonate concentration, SBE = standard base excess, AG = Anion gap, SIDm = measured strong ion difference, A_tot_ = Acid total based on albumin _(Alb)_ or total protein _(Prt),_ SIG = strong ion gap, XA = unmeasured anions, NEFA = nonesterified fatty acid concentration, BHBA = β-hydroxybutyrate concentration,

^a^ = reference values were calculated based on cluster 2 (25% and 75% interquartile ranges),

^b^ = common reference values that were set in literature [39],

Ø = median is within reference values,

↑ / ↓ = median is above / below reference value,

(↑) / (↓) means only relatively increase / decrease, but within the fixed reference values

**Table 3:** Assignment of examined cows to the clusters and their clinical characterization (median values or percentages).

Cluster	1	2	3	4	5	6	7
No. of cows	19	37	27	44	5	10	3
No. of lactating cows (% within the cluster)	-	6	-	1	1	8	-
	-	16.2	-	2.3	20	80	-
No. of farms	4	11	11	12	4	3	1
Days in milk	3	5	6	4	8	54	1
Median age of cows (months)	40.8	41.4	54.2	43.9	56.0	62.3	62.4
Body condition score	3.5	3.25	3.25	3.25	3.25	2.5	4.5
Rectal temperature (°C)	38.7	38.6	38.9	38.7	39.1	38.3	38.7
Category of rumen fill							
1 (%)	52.1	29.7	48.1	40.9	80	20	100
2 (%)	47.9	54.1	48.1	52.3	20	50	0
3 (%)	0	16.2	3.7	6.8	0	30	0

**Cluster 1** included 19 fresh cows, 15 of them from the same farm. They showed slight acidosis in urine as well as in the different approaches of blood gas analysis, while high sodium and chloride ion concentrations were detected in plasma and urine.

**Cluster 2** contained 37 cows, 16% of which were high lactating. No deviations in urine or other metabolism were measured.

**Cluster 3** included 27 fresh cows, which were characterized by an absolute lack of albumin with concurrently increased globulins, so that the concentration of total protein appeared normal.

**Cluster 4** included 44 animals from 12 farms, most of them fresh cows, which showed an acidosis in urine marked by an increased acid excess. This deviation was characterized as strong ion acidosis (decreased SID) in blood, but was also detected with traditional parameters bicarbonate and base excess.

**Cluster 5** contained 5 cows with noticeably increased cBHBA. AG and XA were markedly elevated compared to the other clusters, while SIG_(Alb)_ as well as SIG_(Prt)_ were distinctly lower. Regarding the other parameters in the blood and the clinical results, the cows showed partial, but not uniform deviations.

**Cluster 6** included 10 cows, of which 8 were lactating cows from the same farm. Magnesium and cholesterol concentrations in serum were higher compared to the other clusters. Furthermore, concentrations of gamma-globulins (and therefore total protein) were notably high. The animals showed predominately moderately- and well-filled rumens and the lowest BCS compared to the others.

**Cluster 7** contained only 3 fresh cows from 1 farm. These animals displayed considerably high values for NEFA and bilirubin serum concentration. In addition, lower measured SID, AG and XA as well as higher SIG_Alb_ or SIG_Prt_ were detected in all cows compared to the other clusters.

## Discussion

The aim of the study was to obtain deeper insights into the pathophysiology of acid-base imbalances in metabolically burdened dairy cows despite being clinically healthy. Results of urinary NABE (the routinely used method) for monitoring were completed and compared with acid-base assessment based on blood analysis in dairy herds. We wanted to elucidate whether the traditional approach or the modern strong ion approach provided similar or, in combination, even more information about the acid-base status in cattle. Assuming physiological conditions within clinically healthy animals, acid-base imbalances in blood should be compensated by renal excretion and/or alveolar exchange. Therefore, in routine monitoring of clinically healthy cows, one would expect to identify alterations in the acid-base equilibrium in urine rather than in blood. Despite this theoretical assumption, an evaluation of whether modern strong ion theory was superior in detecting and characterizing subclinical metabolic changes compared to the traditional approach was required. Fresh cows with expected variations in protein supplementation as a result of feeding alterations in the transition period, colostrum production and inadequate feed intake were mainly included in the study to verify the influence of serum protein on acid-base balance in general and on the the strong ion gap (SIG) as described by strong ion theory, in particular [7–9].

Since the present study was designed to facilitate the generation of hypotheses, an exploratory data analysis seemed to provide the best summary of the heterogeneous raw data obtained. A cluster analysis was conducted to identify groups of cows with similar physiological status based on several diagnostic variables. The 7 clusters emerged revealed an acceptable internal validity. We omitted further (inferential) statistical analyses because we did not want our exploratory results to appear more generally valid than they were.

We evaluated and compared the results of urine, traditional blood gas analysis and the strong ion approach within and between the 7 clusters. According to the recommendations of Kimura et al. [5], laboratory specific reference values were determined. Cluster 2 showed no deviations in urine as well as in the other metabolic parameters in blood (interpreted with common reference values [39]). Consequently, it was set as the metabolically healthy reference sample. For the parameters of acid base status in urine as well as traditional and modern approach for blood gas analysis, the 25% and 75% interquartile ranges of cluster 2 were determined as the lower and upper internal “within-study” reference values (Table 2).

When evaluating clinical behavior and general metabolic parameters, the influence of lactation stage was clearly noticeable. Cluster 6, which contained almost exclusively high lactating cows, showed the lowest BCS, best proportional rumen fill and, with normal NEFA and BHBA values, no detectable energy deficit. Serum cholesterol concentrations were notably higher in cluster 6 compared to the fresh cow clusters and showed a good relation to DIM, as previously described [40]. In contrast to this, the other clusters (except cluster 2 which also contained lactating cows) showed increased NEFA concentrations in serum, as expected for fresh cows. Likewise, we could see deviations of albumin and γ-globulin concentrations as the two important protein fractions. GLDH-activity and urea concentration in blood, as well as calcium and magnesium concentrations in urine, were within the common reference ranges and showed no distinct variations between the clusters. Analyzing the clusters in general, clusters 5 and 7 obviously contained a few animals which appeared to be metabolically sick. They showed excessively increased NEFA or BHBA concentrations. Therefore, the general interpretation of the results of the cluster analysis was done without these two deviant clusters. These two clusters needed to be considered separately from the others.

In urine, diverse kinds of acid-base disturbances were observed. Besides the two clusters that did not show remarkable deviations in acid-base status (clusters 1 and 2), we diagnosed two acid-burdened clusters (clusters 3 and 4) and one alkaline-stressed cluster (cluster 6).

The traditional blood gas analysis showed some agreement with the results of urine analysis in this study. One cluster with strongly acidic urine (cluster 3) and even one alkaline cluster (6) showed no abnormalities in the traditional approach. With respect to the visibly healthy cows which were sampled in routine herd monitoring, we didn’t expect extensive deviations in blood acid-base balance. With the demonstrated renal compensation, the blood pH was kept at a constant level. A limitation of the traditional approach is its inability to differentiate between various possible reasons for metabolic disturbances [12]. The AG seemed unsuitable for providing any crucial further information about the clusters. Lowered AG in two of the clusters, representing increased unidentified cations or decreased unidentified anions [11], did not contribute to the detected acidosis in urine and could not be explained by the authors. In fact, there is an accordance of low AG with increased NEFA in the clusters (clusters 3 and 4) which needs further investigation. In addition, the dependence of AG on protein (particularly albumin) concentration could be assumed as described previously [6, 7]. Obviously, low blood concentrations of albumin contributed to low AG in cluster 3.

Unfortunately, most conventional laboratory blood gas analyzers do not actually titrate samples to determine standard base excess but approximate the value by various equations that usually assume a constant protein concentration. Since the protein concentration has marked effects on buffer capacity, this can lead to errors in certain clinical situations with changes in serum protein levels. Using the strong ion approach the variables SID and A_tot_ have been proposed for a more intensive evaluation of the acid-base status [12]. A metabolic acidosis is characterized by a decrease in strong ions (SID) or an increase in non-volatile buffer ions (A_tot_), while an increase in SID or a decrease in A_tot_ indicates a metabolic alkalosis [8]. By using the strong ion approach parameters, we observed higher differentiated outcomes, which corresponded slightly better to the results obtained by urine analysis. In general, the acid base assessment of the clusters based on the two different approaches of blood gas analysis corresponded in only 2 of the 4 evaluated clusters. Depending on the protein fraction used to determine A_tot_, several irregularities in acid-base status were observed within the clusters. The SID was calculated on the basis of 3 formulae which were expanded with additional ion fractions. Measured SIDs with 3, 4 and 5 ion fractions had minor differences and responded similarly within the clusters.

For a profound evaluation of the study, the clusters should be discussed individually across the different methods.

**Cluster 2**, used as within-study reference, was characterized by a higher NABE and base-acid ratio in urine compared to most of the other clusters. Although both variables were within the common reference ranges [39], they showed a tendency to alkalosis. In venous blood, high levels of bicarbonate and base excess were measured by blood gas analysis compared to the other clusters. However, an alkaline metabolism is physiological for herbivores like cattle [19], thus it can be assumed that the animals of cluster 2 were healthy and had a good appetite, as they had predominately moderately or well filled forestomachs.

**Cluster 1** contained apparently healthy fresh cows with slight acidosis according to the results of the traditional approach and urine measurement. The decreased SID indicated an acidosis, too. The increases in plasma concentrations of sodium and chloride ions as well as their raised excretion in urine were probably caused by an inflated dose of salt in the feedlot and reflected most likely farm-specific feeding components. Since cluster 1 consisted of early fresh cows with an average of only 3 days in milk, the relative lack of γ-globulins, and therefore also of total protein, can be interpreted as a normal deficit due to colostrum production [41]. The low total protein concentrations in blood led to a slight non-volatile buffer ion alkalosis (Atot–alkalosis) in the strong ion approach with a minor reduced SIG _(Prt)_, but without visible effects on the acid-base balance. The slight reduction of SIG_(Prt)_ should not be interpreted as an increase of unmeasured strong anions in this case because it was the result of the lack of γ-globulins. Overall, the SIG_(Alb)_ and XA were not visibly deviant. It can be suggested that use of albumin instead of total protein would be better for determining unmeasured ions in early fresh cows.

The cows summarized in **cluster 3** showed a strong acidosis determined by urine measurement, but no aberrations in the traditional parameters, i.e. blood bicarbonate concentration and base excess. Low albumin concentrations and A_tot(Alb)_ values in blood were obtained. Together with increased globulin fractions, these deviations could be interpreted as an expected acute phase response after calving with suppressed feed intake. The animals were 6 days in lactation on average, i.e. when regenerative processes in the uterus and birth canal are at a high level. In this group, the mean rectal temperature tended to be higher compared to the others, which also indicated inflammatory and/or tissue repairing processes. In addition to low albumin, low concentration of Mg^2+^ and L-lactate were observed, while rumen fill was mostly moderate or nearly empty. Consequently, a reduced feed intake and basal metabolism could be assumed. The obvious acidosis in urine, marked by decreased pH, NABE and base-acid ratio, as well as the low measured SIDs, which also identified acidosis, were in agreement with a low feed intake. It could be concluded that the acid load was probably compensated by the lack of albumin. Therefore, no acidosis was diagnosed with the traditional blood gas parameters, while the urine parameters as well as the modern approach with SID and A_tot(Alb)_ identified this mixed metabolic imbalance more accurately.

**Cluster 4** was mainly made up of normal, healthy fresh cows with reduced feed intake, defined by predominately moderately filled or nearly empty forestomachs. In contrast to the animals in cluster 3, the blood concentrations of albumin were normal. The reduced feed intake presumably led to an acidic load, represented by markedly lower SIDs in the strong ion approach, and also showed lower pH, bicarbonate and base excess in the traditional approach when compared to other clusters. In the urine, the renal compensation of the detected acid load was obviously noticeable as an increase in acid excess represented by low NABE and base-acid ratio. The urine-based diagnosis as well as the reduced SID data measured in blood were in good agreement with evaluation of the acid-base status by traditional parameters in this cluster.

**Cluster 6** almost included high lactating cows from only one farm. Consequently, farm-specific effects need to be discussed. We assumed a good food intake based on well-filled forestomachs, as well as by high concentrations of magnesium and cholesterol and low NEFA and BHBA concentrations in the blood. The high sodium excretion in urine suggested a mineral burden in the feedlot, which can probably be explained by elevated doses of sodium bicarbonate (used for rumen buffering on this particular farm). Extensive mineral supplementation may lead to an elevated base excess which is compensated by the kidneys and can be measured by high NABE, base-acid ratio and base excess in urine. Due to effective renal elimination of sodium, no aberrations were seen in the traditional parameters, i.e. bicarbonate and base excess as well as in the measured SID in blood. The lower hematocrit level compared to the other clusters (high lactation vs. fresh cows) might be a result of the progressive lactation as described by others [42]. Enquiries on the farm revealed an ongoing ringworm infection in the dairy livestock over the examination period which could induce an antibody response [43] and may explain the high concentrations of γ-globulins. The increased γ-globulin levels led to a moderate non-volatile buffer ion acidosis (A_tot(Prt)_-acidosis), which was not reflected in the traditional acid-base parameters because the traditional approach is limited to normal temperature, pH, protein concentration and sodium concentration. Consequently, the application of this traditional approach for describing the acid-base balance in animals with protein aberrations may lead to false results [11]. Indeed, we most likely identified a mixed metabolic disturbance on this farm caused by a high sodium load and increased protein amounts.

Describing and interpreting **cluster 5** is difficult, because it contained only five (although very different) cows presenting similarities in a few important parameters. Primarily, the animals showed high BHBA values. The ketone bodies, classified as unmeasured anions, were reliably detected with the modern approaches by increased XA and/or decreased SIG_(Alb)_ and SIG_(Prt)_. This was in accordance with other publications, where these trends of deviation were also described for an excess of unmeasured anions like BHBA [11, 14]. In contrast to this, the traditional AG was increased when evaluated with internal reference values, but remained within the suggested reference range for adult cattle [33]. Animals in cluster 5 showed the lowest pCO_2_ data in venous blood (S1 Table). This effect could be interpreted as a respiratory compensation for the acid burden and has also been described in humans [44]. The acidosis in urine, measured by decreased NABE and base-acid ratio and especially increased ammonium excretion, showed the renal compensation of the acid load [2] provided by ketogenic metabolism. Inadequate feed intake reinforces the acidotic stress. Except for the single high lactating cow in this cluster, the other fresh cows presented markedly lower concentrations of glucose, L-lactate, cholesterol and albumin, and also high NEFA concentrations in the blood. Within the modern approach, the lack of albumin led to an A_tot_-alkalosis. Additionally, in comparison to the other clusters, we observed higher measured SID data (usually also interpreted as alkalosis). Chloride ion concentrations in the plasma were also low, probably as a compensation mechanism against the acid load. Likewise, it is possible that these ketotic cows had early stages of displaced abomasum with intestinal chloride retention. Taking only the traditional parameters for acid base assessment into account, i.e. bicarbonate and base excess, only an acidosis was identified.

**Cluster 7** included only 3 cows and, as for cluster 5, the results must be interpreted carefully. Since outliers will have a strong effect on the results, the three animals need to be considered separately. In addition, sampling time was close to calving so that aberrations in metabolism, e.g. low calcium concentrations, were expected. The urine parameters NABE and base-acid ratio tended to alkalosis, although blood pH, bicarbonate and base excess in the blood showed no uniform deviations. Alkalotic load may have been caused by reduced liver function due to fatty acid overload, which resulted in impaired ureagenesis and therefore increased ammonium concentrations in the blood. Our findings are in agreement with results published by others [45, 46]. Indeed, only 1 of the 3 cows showed high bicarbonate concentrations, and none of the cows had increased urinary ammonium excretion. However, a notable decrease in measured SID, leading to a strong ion acidosis, was detected in all cows. Perhaps this, together with higher albumin concentration compared to the other clusters, is a metabolic compensation for the alkaline burden resulting from the ammonia. A high level of unmeasured anions would be expected, because NEFA belong to the latter [14]. Confusingly, low AG and XA, as well as high SIGs, indicated an increase in unmeasured cations or a decrease in unmeasured anions. This interrelation needs further investigation. The high concentrations of chloride, which primarily led to the decreased SID and therefore to the calculated low AG and high SIG, should be questioned critically. In a previous study, high chloride values and low AGs were documented as measurement errors in patients with hyperlipidemia [47]. Although they described another measurement method, this error should be also considered.

In view of the diversity of the outcomes of this work, the weakness of the study has to be discussed carefully. The selected animals for the study represent neither a herd average nor a randomized sample. Obviously diseased cows were excluded and the distribution over different lactation stages was inhomogeneous with predominantly fresh cows immediately after calving. Furthermore, the allocation of the farms varied between 4 and 48 cows per farm. Therefore, no general valid recommendations can be given for other herds, and further investigations including a larger number of animals, other farms and different lactation stages are necessary. To minimize suffering for cows, we only were capable to take venous blood samples. By using venous blood for evaluating acid base status, only the metabolic component of acid base disturbances could be assessed reliably, but there was no possibility to determine any valid evaluation of pulmonary gas exchange [48]. Therefore, no general valuation of the respiratory component of acid base status was done within the clusters. The lack of reference values for the strong ion theory parameters produced difficulties in the unprejudiced interpretation of the clusters. For future analysis, thresholds should be determined for adult cattle. In addition, a consistent use of either albumin or total protein for determining A_tot_ and SIG is important for future studies. Depending on whether globulins are included or not, varied interpretations of the amount of unmeasured ions are possible (S1 Table). In this study, the SIG_(Alb)_, calculated on the basis of albumin concentration, corresponded well with the variable XA as well as with clinical and general metabolic conditions when compared to the SIG_(Prt)_ (Table 2). Indeed, the SIG_(Prt)_ and the AG showed some deviations that could not be explained altogether. In contrast, the SIG_(Alb)_ and even more the XA demonstrated the advantages of strong ion approach in identifying unmeasured ions.

## Conclusions

The parameters of the strong ion difference theory, particularly SID_m_, A_tot_ and SIG_Alb_ or XA, provided more in-depth information about acid-base status, especially in cases of subclinical acid-base disorders compared to the traditional parameters base excess and bicarbonate in blood. Consequently, the strong ion approach seems to be a suitable supplement for monitoring acid-base balance in dairy cattle. The acid-base status of fresh cows with protein aberrations in blood could be differentiated in a much better way using the strong ion approach than by traditional blood gas analysis or by the measurement of urinary excretion. Further investigations are necessary to obtain reference values and standardized calculation formulae for the strong ion parameters. Additionally, the influence of several feeding components, such as anionic salts or rumen buffers, on strong ion parameters should be taken into account in further research.

## Supporting information

S1 Table Results (medians, first and third quartiles) of all measured and calculated parameters within the 7 generated clusters. (DOCX)
