# Supplementary material for: Acid-base assessment of post-parturient German Holstein dairy cows from jugular venous blood and urine: A comparison of the strong ion approach and traditional blood gas analysis
Source: PLoS One. 2019 Jan 16;14(1):e0210948. doi: 10.1371/journal.pone.0210948 (PMC6334950; doi:10.1371/journal.pone.0210948)
Supplement: S1 Table — (DOCX) [file pone.0210948.s001.docx]

S1 Table.

|  |  | **Cluster 1** | | | **Cluster 2** | | | **Cluster 3** | | | **Cluster 4** | | | **Cluster 5** | | | **Cluster 6** | | | **Cluster 7** | | |
| --- | --- | --- | --- | --- | --- | --- | --- | --- | --- | --- | --- | --- | --- | --- | --- | --- | --- | --- | --- | --- | --- | --- |
| **Variables** | **Units** | **Q I** | **Median** | **Q III** | **Q I** | **Median** | **Q III** | **Q I** | **Median** | **Q III** | **Q I** | **Median** | **QIII** | **Q I** | **Median** | **Q III** | **Q I** | **Median** | **Q III** | **Q I** | **Median** | **Q III** |
| **pH (u)** |  | 8.09 | 8.17 | 8.29 | 8.17 | 8.31 | 8.35 | 7.03 | 7.83 | 8.06 | 7.79 | 8.14 | 8.24 | 6.88 | 7.88 | 7.90 | 8.31 | 8.33 | 8.34 | 8.40 | 8.43 | 8.43 |
| **cNABE (u)** | mmol/l | 81.50 | 119.00 | 137.50 | 106.00 | 165.00 | 210.00 | 5.00 | 40.00 | 101.00 | 39.00 | 85.50 | 135.00 | -27.00 | 43.00 | 56.00 | 212.75 | 226.00 | 243.75 | 180.50 | 194.00 | 209.00 |
| **BAR (u)** | mmol/l | 1.77 | 2.24 | 2.83 | 2.29 | 3.05 | 3.54 | 1.10 | 1.46 | 2.01 | 1.31 | 1.85 | 2.32 | 0.83 | 1.45 | 1.49 | 4.07 | 4.44 | 5.14 | 4.67 | 4.88 | 5.14 |
| **cBE (u)** | mmol/l | 150.00 | 205.00 | 245.00 | 195.00 | 245.00 | 280.00 | 110.00 | 170.00 | 210.00 | 158.75 | 205.00 | 255.00 | 130.00 | 130.00 | 170.00 | 266.25 | 300.00 | 327.50 | 230.00 | 250.00 | 262.50 |
| **cAE (u)** | mmol/l | 70.50 | 92.00 | 97.00 | 60.00 | 79.00 | 90.00 | 72.00 | 101.00 | 131.50 | 76.25 | 105.50 | 136.25 | 83.00 | 97.00 | 120.00 | 52.00 | 62.50 | 74.00 | 45.50 | 50.00 | 52.50 |
| **cAmm (u)** | mmol/l | 2.50 | 4.00 | 7.00 | 2.00 | 3.00 | 6.00 | 5.00 | 7.00 | 17.00 | 5.00 | 8.00 | 12.25 | 8.00 | 16.00 | 35.00 | 2.00 | 3.00 | 3.75 | 1.00 | 1.00 | 1.50 |
| **cNa (u)** | mmol/l | 51.80 | 104.00 | 154.40 | 9.70 | 53.80 | 101.30 | 5.00 | 5.00 | 33.35 | 5.00 | 13.60 | 56.75 | 5.00 | 8.10 | 25.70 | 119.25 | 134.70 | 162.03 | 51.70 | 52.50 | 73.45 |
| **cK (u)** | mmol/l | 172.75 | 221.80 | 262.85 | 125.80 | 188.70 | 230.80 | 112.30 | 168.40 | 249.15 | 177.33 | 215.85 | 251.28 | 89.60 | 140.80 | 151.80 | 97.55 | 130.65 | 175.45 | 107.60 | 118.50 | 142.30 |
| **cMg (u)** | mmol/l | 7.33 | 11.35 | 16.94 | 5.14 | 8.54 | 11.94 | 4.31 | 9.66 | 15.27 | 7.01 | 8.56 | 17.21 | 8.15 | 8.39 | 9.03 | 6.14 | 9.17 | 11.77 | 3.24 | 4.59 | 8.69 |
| **cCa (u)** | mmol/l | 0.50 | 0.50 | 3.48 | 0.50 | 0.50 | 0.57 | 0.50 | 0.67 | 2.61 | 0.50 | 0.64 | 2.63 | 0.50 | 0.50 | 1.04 | 0.50 | 0.50 | 0.50 | 0.50 | 0.50 | 0.50 |
| **cCl (u)** | mmol/l | 139.50 | 149.00 | 183.00 | 19.00 | 38.00 | 65.00 | 15.00 | 46.00 | 61.50 | 17.75 | 40.00 | 105.00 | 15.00 | 15.00 | 17.00 | 16.50 | 21.50 | 32.50 | 15.00 | 18.00 | 30.50 |
| **cBHBA** | µmol/l | 509.50 | 716.00 | 1119.50 | 564.00 | 692.00 | 812.00 | 581.00 | 758.00 | 933.00 | 696.25 | 821.00 | 987.75 | 1730.00 | 2366.00 | 2449.00 | 592.50 | 610.50 | 657.25 | 794.50 | 904.00 | 1005.50 |
| **cCholesterol** | mmol/l | 1.38 | 1.88 | 2.44 | 1.68 | 2.41 | 2.79 | 1.64 | 1.80 | 2.22 | 1.84 | 2.20 | 2.76 | 1.72 | 1.80 | 2.07 | 4.91 | 5.93 | 6.50 | 1.63 | 2.24 | 2.28 |
| **cGlucose** | mmol/l | 2.93 | 3.35 | 3.78 | 3.20 | 3.30 | 3.70 | 3.18 | 3.50 | 3.75 | 3.05 | 3.53 | 3.83 | 2.20 | 2.90 | 3.15 | 3.35 | 3.53 | 3.64 | 3.28 | 3.55 | 3.83 |
| **cL-lactate** | mmol/l | 0.48 | 0.55 | 0.93 | 0.50 | 0.60 | 0.80 | 0.40 | 0.50 | 0.70 | 0.50 | 0.68 | 1.15 | 0.45 | 0.50 | 0.85 | 0.53 | 0.65 | 0.69 | 0.48 | 0.55 | 0.75 |
| **cBilirubin** | µmol/l | 3.74 | 4.74 | 7.32 | 2.88 | 3.58 | 4.48 | 4.41 | 7.43 | 11.13 | 4.29 | 5.88 | 8.80 | 7.49 | 7.68 | 8.88 | 2.06 | 2.28 | 4.06 | 19.74 | 25.90 | 27.91 |
| **aGLDH** | nkat/l | 88.05 | 145.80 | 181.05 | 142.90 | 197.70 | 310.30 | 124.05 | 158.00 | 273.10 | 141.50 | 200.00 | 258.15 | 171.20 | 319.80 | 341.10 | 149.18 | 188.75 | 236.58 | 78.90 | 79.60 | 103.95 |
| **cUrea** | mmol/l | 2.70 | 3.50 | 4.00 | 3.20 | 3.80 | 4.40 | 2.25 | 2.90 | 3.55 | 3.28 | 3.90 | 4.75 | 3.00 | 4.80 | 5.00 | 3.88 | 4.30 | 4.40 | 4.15 | 4.20 | 4.25 |
| **cProtein total** | g/l | 63.15 | 68.80 | 70.90 | 68.10 | 72.10 | 75.60 | 71.25 | 74.10 | 78.10 | 68.43 | 73.85 | 78.60 | 61.50 | 72.30 | 75.40 | 83.73 | 87.70 | 88.43 | 70.30 | 74.20 | 79.95 |
| **cAlbumin** | g/l | 27.85 | 31.40 | 32.25 | 28.20 | 31.60 | 33.60 | 26.05 | 27.70 | 29.35 | 31.65 | 33.55 | 35.08 | 26.00 | 27.90 | 32.10 | 30.00 | 33.90 | 35.23 | 35.50 | 35.80 | 36.45 |
| **cγ-Globulin** | g/l | 14.55 | 17.40 | 19.70 | 17.50 | 21.60 | 24.10 | 22.30 | 27.80 | 30.25 | 17.78 | 22.20 | 23.98 | 15.80 | 23.40 | 26.20 | 29.05 | 31.05 | 39.80 | 18.60 | 22.70 | 26.65 |
| **cα1-globuline** | g/l | 10.00 | 10.80 | 12.30 | 10.80 | 11.60 | 12.70 | 11.50 | 12.60 | 13.10 | 10.00 | 10.95 | 11.85 | 11.20 | 11.30 | 11.60 | 11.13 | 11.50 | 12.53 | 9.10 | 9.50 | 9.55 |
| **cα2-globuline** | g/l | 2.85 | 3.50 | 4.90 | 2.90 | 3.10 | 3.80 | 3.50 | 4.10 | 4.65 | 2.58 | 3.00 | 3.40 | 3.30 | 3.80 | 4.20 | 2.73 | 3.15 | 3.38 | 2.45 | 2.60 | 2.65 |
| **cβ-globuline** | g/l | 4.15 | 4.60 | 4.85 | 4.10 | 4.60 | 5.20 | 3.45 | 4.00 | 4.70 | 3.90 | 4.40 | 4.93 | 3.00 | 3.50 | 3.70 | 3.93 | 4.35 | 5.10 | 4.65 | 4.80 | 5.30 |
| **cNa^+^** | mmol/l | 140.50 | 142.00 | 143.00 | 138.00 | 140.00 | 142.00 | 138.00 | 139.00 | 140.75 | 138.00 | 140.00 | 141.00 | 135.50 | 138.00 | 142.00 | 139.25 | 140.00 | 141.00 | 142.25 | 142.50 | 142.75 |
| **cMg** | mmol/l | 1.05 | 1.19 | 1.23 | 1.14 | 1.29 | 1.45 | 0.92 | 1.00 | 1.19 | 1.07 | 1.20 | 1.36 | 1.05 | 1.12 | 1.12 | 1.23 | 1.31 | 1.39 | 1.25 | 1.29 | 1.35 |
| **cCl^-^** | mmol/l | 102.25 | 105.00 | 105.25 | 97.00 | 100.00 | 103.00 | 101.00 | 102.00 | 104.00 | 100.50 | 102.25 | 105.00 | 97.50 | 98.00 | 100.00 | 100.63 | 102.00 | 104.13 | 108.00 | 108.50 | 109.25 |
| **cK^+^ (b)** | mmol/l | 3.80 | 4.00 | 4.28 | 3.70 | 3.90 | 4.15 | 3.78 | 3.90 | 4.15 | 3.75 | 3.95 | 4.10 | 3.90 | 4.10 | 4.20 | 3.70 | 3.80 | 3.98 | 4.05 | 4.15 | 4.20 |
| **cCa^2+^ (b)** | mmol/l | 1.17 | 1.19 | 1.28 | 1.15 | 1.20 | 1.25 | 1.18 | 1.23 | 1.26 | 1.16 | 1.22 | 1.26 | 1.14 | 1.20 | 1.20 | 1.21 | 1.23 | 1.27 | 1.05 | 1.10 | 1.11 |
| **cPi (b)** | mmol/l | 1.41 | 1.58 | 2.14 | 1.55 | 1.76 | 1.99 | 1.36 | 1.53 | 1.77 | 1.43 | 1.58 | 1.88 | 1.76 | 1.83 | 1.84 | 1.13 | 1.37 | 1.55 | 1.02 | 1.33 | 1.33 |
| **pH (v)_BT_** |  | 7.37 | 7.38 | 7.40 | 7.38 | 7.39 | 7.40 | 7.37 | 7.40 | 7.41 | 7.35 | 7.38 | 7.39 | 7.36 | 7.38 | 7.40 | 7.38 | 7.39 | 7.41 | 7.39 | 7.40 | 7.41 |
| **cHCO_3_^-^** | mmol/l | 26.88 | 27.78 | 28.84 | 28.20 | 29.36 | 30.88 | 26.91 | 28.83 | 29.29 | 26.56 | 27.76 | 28.84 | 26.35 | 26.81 | 27.42 | 26.39 | 28.31 | 28.96 | 26.98 | 26.99 | 28.13 |
| **cHCO_3_^-^ (st)** | mmol/l | 25.70 | 26.65 | 27.50 | 26.95 | 27.85 | 28.80 | 26.40 | 27.45 | 28.15 | 25.24 | 26.30 | 27.28 | 26.15 | 26.60 | 26.90 | 26.16 | 27.13 | 28.13 | 26.58 | 27.00 | 27.73 |
| **cBase** | mmol/l | 2.28 | 3.30 | 4.53 | 3.75 | 4.75 | 6.05 | 3.08 | 4.10 | 5.08 | 2.03 | 3.03 | 4.16 | 3.00 | 3.05 | 3.10 | 2.64 | 4.03 | 4.94 | 3.15 | 3.60 | 4.33 |
| **cBase (Ecf)** | mmol/l | 2.43 | 3.70 | 4.80 | 4.25 | 5.30 | 6.50 | 3.20 | 4.75 | 5.40 | 2.33 | 3.63 | 4.65 | 2.95 | 3.10 | 3.25 | 2.74 | 4.33 | 5.23 | 3.38 | 3.85 | 4.63 |
| **pCO_2 (BT)_** | kPa | 6.52 | 6.62 | 6.73 | 6.66 | 6.84 | 7.13 | 6.29 | 6.49 | 6.89 | 6.35 | 6.71 | 6.96 | 5.77 | 6.06 | 6.56 | 6.23 | 6.57 | 6.76 | 6.11 | 6.42 | 6.53 |
| **AG** | mmol/l | 12.94 | 14.20 | 14.95 | 13.49 | 14.00 | 15.25 | 11.93 | 12.41 | 13.12 | 11.95 | 13.10 | 14.08 | 16.66 | 17.18 | 18.15 | 13.99 | 14.08 | 14.50 | 9.57 | 10.16 | 10.82 |
| **SIDm_3_** | mmol/l | 40.43 | 41.70 | 42.98 | 42.05 | 43.55 | 45.35 | 39.63 | 41.00 | 41.58 | 39.50 | 40.80 | 41.81 | 41.90 | 44.60 | 46,95 | 40.84 | 42.23 | 43.09 | 37.70 | 38.25 | 38.35 |
| **SIDm_4_** | mmol/l | 39.53 | 41.10 | 42.38 | 41.35 | 42.95 | 44.40 | 39.00 | 40.55 | 41.18 | 38.56 | 40.00 | 40.93 | 41.45 | 43.75 | 45.55 | 40.05 | 41.55 | 42.56 | 37.13 | 37.50 | 37.60 |
| **SIDm_5_** | mmol/l | 40.71 | 42.42 | 43.59 | 42.60 | 44.22 | 45.47 | 40.18 | 41.71 | 42.36 | 39.70 | 41.23 | 42.15 | 42.69 | 44.82 | 46.75 | 41.31 | 42.80 | 43.82 | 38.19 | 38.51 | 38.65 |
| **A_tot(Alb)_** | mmol/l | 21.17 | 23.86 | 24.51 | 21.43 | 24.02 | 25.54 | 19.80 | 21.05 | 22.31 | 24.05 | 25.50 | 26.66 | 19.76 | 21.20 | 24.40 | 22.80 | 25.76 | 26.77 | 26.98 | 27.21 | 27.70 |
| **A_tot(Prt)_** | mmol/l | 22.73 | 24.77 | 25.52 | 24.52 | 25.96 | 27.22 | 25.65 | 26.68 | 28.12 | 24.63 | 26.59 | 28.30 | 22.14 | 26.03 | 27.14 | 30.14 | 31.57 | 31.83 | 25.31 | 26.71 | 28.78 |
| **SIG_(Alb)_** | mmol/l | 0.42 | 1.78 | 2.68 | 0.52 | 2.18 | 3.39 | 0.50 | 2.08 | 3.71 | 2.64 | 4.18 | 5.92 | -3.64 | -3.51 | -2.85 | 1.30 | 3.30 | 4.50 | 7.74 | 8.85 | 9.63 |
| **SIG_(Prt)_** | mmol/l | 0.95 | 2.10 | 3.77 | 2.39 | 3.64 | 4.69 | 4.64 | 6.63 | 7.56 | 3.82 | 5.36 | 6.44 | -1.81 | -1.72 | 0.66 | 6.27 | 7.58 | 8.00 | 6.61 | 8.50 | 10.36 |
| **cXA** | mEq/l | 7.52 | 8.49 | 9.18 | 7.39 | 8.17 | 8.78 | 4.71 | 6.68 | 7.79 | 4.73 | 6.08 | 7.52 | 12.46 | 12.58 | 12.89 | 5.39 | 6.06 | 7.08 | 0.44 | 2.04 | 3.09 |
| **BT** | °C | 38.50 | 38.70 | 38.95 | 38.40 | 38.60 | 38.90 | 38.55 | 38.90 | 39.30 | 38.40 | 38.70 | 38.90 | 38.70 | 39.10 | 39.40 | 37.93 | 38.25 | 38.50 | 38.60 | 38.70 | 39.10 |
| **BCS** |  | 3.25 | 3.50 | 3.75 | 3.00 | 3.25 | 3.50 | 3.00 | 3.25 | 3.50 | 3.00 | 3.25 | 3.75 | 2.75 | 3.25 | 3.50 | 2.31 | 2.50 | 3.19 | 4.00 | 4.50 | 4.50 |
| **DIM** |  | 1.00 | 3.00 | 4.00 | 3.00 | 5.00 | 9.00 | 5.00 | 6.00 | 9.50 | 2.75 | 4.00 | 8.00 | 4.00 | 8.00 | 14.00 | 43.00 | 53.50 | 58.25 | 1.00 | 1.00 | 1.50 |
| **Hct** | l/l | 0.35 | 0.36 | 0.38 | 0.34 | 0.36 | 0.37 | 0.32 | 0.34 | 0.36 | 0.36 | 0.38 | 0.40 | 0.30 | 0.33 | 0.36 | 0.29 | 0.30 | 0.31 | 0.39 | 0.39 | 0.40 |

Legend to S1 Table

Q I = first quartile / Q III = third quartile of values within the cluster, pH (u) = pH-level in urine, cNABE (u) = net acid-base excretion in urine, BAR (u) = base-acid ratio in urine, cBE (u) = base extent in urine, cAE (u) = acid extent in urine, cAmm (u) = concentration of ammonia in urine, cNa^+^ (u) = sodium ion concentration in urine, cK^+^ (u) = potassium ion concentration in urine, cMg (u) = magnesium concentration in urine, cCa^2+^ (u)  = calcium ion concentration in urine, cCl^-^ (u)  = chloride ion concentration in urine, cBHBA = concentration of β-hydroxybutyrate in serum, aGLDH = activity of glutamate-dehydrogenase in serum, cNa^+^ (b) = sodium ion concentration in plasma, cMg (b) = magnesium concentration in serum, cCl^-^(b) = chloride ion concentration in plasma, cK^+^(b) = potassium ion concentration in plasma, cCa^2+^ (b)  = calcium ion concentration in plasma, cPi (b)  = concentration of inorganic phosphorous in serum, pH (v)_BT_ = venous pH-level in plasma, corrected for rectal measured body temperature, cHCO_3_^-^ = actual bicarbonate concentration in plasma, cHCO_3_^-^ (st)  = standard bicarbonate concentration, cBase = actual base excess in plasma, cBase (Ecf)  = standard base excess, pCO_2 (BT)_ = partial pressure of carbon dioxide, corrected for body temperature, AG = anion gap, SIDm_3/4/5_ = measured strong ion difference, A_tot(Alb)_  = acid total (calculated with albumin), A_tot(Prt)_  = acid total (calculated with total protein), SIG_(Alb)_ = strong ion difference (calculated with albumin), SIG_(Prt)_  = strong ion difference (calculated with total protein), XA = unmeasured anions, BT = rectal measured body temperature, BCS = body condition score, DIM = no. of days in milk, Hct = hematocrit
